# Sampling Methods for Luminescence Dating of Subsurface Deposits from Cores

**DOI:** 10.3390/mps2040088

**Published:** 2019-11-22

**Authors:** Michelle Nelson, Tammy Rittenour, Harriet Cornachione

**Affiliations:** 1 USU Luminescence Laboratory, North Logan, UT 84341, USA; tammy.rittenour@usu.edu; 2 Department of Geosciences, Utah State University, Logan, UT 84322-4505, USA; harriet.cornachione@aggiemail.usu.edu

**Keywords:** luminescence sampling, sediment cores, augered sediments, portable dark room

## Abstract

Study of subsurface deposits often requires coring or drilling to obtain samples for sedimentologic and geochemical analysis. Geochronology is a critical piece of information for stratigraphic correlation and rate calculations. Increasingly, luminescence dating is applied to sediment cores to obtain depositional ages. This paper provides examples and discussion of guidelines for sampling sediment core for luminescence dating. Preferred protocols are dependent on the extraction method, sedimentology, core integrity, and storage conditions. The methods discussed include subsampling of sediment in opaque core-liners, cores without liners, previously open (split) cores, bucket auger samples, and cuttings, under red lighting conditions. Two important factors for luminescence sampling of sediment core relate to the integrity of the natural luminescence signal and the representation of the dose rate environment. The equivalent dose sample should remain light-safe such that the burial dose is not reset (zeroed) by light exposure. The sediment sampled for dose rate analyses must accurately represent all units within at least 15 cm above and below the equivalent dose sample. Where lithologic changes occur, units should be sampled individually for dose rate determination. Sediment core extraction methods vary from portable, hand-operated devices to large truck- or vessel-mounted drill rigs. We provide recommendations for luminescence sampling approaches from subsurface coring technologies and downhole samplers that span shallow to deep sample depths.

## 1. Introduction

Luminescence dating is a technique that provides an age estimate for the last time sediment or cultural material was exposed to sunlight or high heat which resets the luminescence signal [1]. Luminescence ages are calculated by dividing the equivalent dose of radiation the sample received during burial (D_E_) by the dose rate environment of the surrounding sediments (D_R_). Special sampling and handling methods are required for luminescence samples to prevent light exposure. Routinely, this involves a light-proof metal, black polyvinyl chloride (PVC), or polyethylene (PE) tube that is pounded horizontally into an exposure of sediments. The collection of sediment surrounding the D_E_ sample for D_R_ calculation is equally important for accurate age determination. Exposures of sediment from erosional escarpments and human-made excavations (i.e., roadcuts, trenches, soil pits) are commonly limited in availability, requiring the use of mechanical collection of cores through augering and drilling to characterize, describe, and date buried stratigraphy. Literature describing the best practices for luminescence sampling are mostly focused on settings where samples can be collected from exposures [2]. Guidelines for luminescence sampling related to core or auger samples are limited, although specific sediment coring technology and luminescence sampling of sand dunes has been introduced [3]. Additionally, subsampling of stored Ocean Drilling Program sediment cores for luminescence dating has also been well-described [4]. Here, we build on these contributions and describe best practices for collection of luminescence samples from a range of core and subsurface sediment collection methods. We also review common coring methods (augering, drilling) and how they should be outfitted to protect the natural luminescence signals. 

Luminescence dating utilizes trapped charge (electrons) that accumulate in defects in quartz or feldspar minerals (sand or silt grain) due to exposure to ionizing radiation to calculate the last time that sediment was exposed to sunlight or heat [1,5]. Following burial or removal from heat, the grain acquires trapped charge proportional to the duration of burial and the radioactivity of the surrounding sediments, plus incident cosmic radiation [1]. The applicable age range for luminescence dating is dependent on the dose rate conditions and mineral properties and typically ranges from 100 years to ≥200,000 years for optically stimulated luminescence (OSL) dating of quartz and up to 500,000 years for infrared stimulated luminescence (IRSL) dating of potassium feldspar [6], however older ages can be obtained using other luminescence methods [7]. OSL and IRSL are advantageous in many settings given that quartz and feldspar are present in most surficial deposits. Moreover, these methods directly relate time with sediment deposition, unlike other methods that use radiometric decay of affiliated material (e.g., radiocarbon dating of charcoal). 

Important factors for reducing uncertainty in luminescence dating are adequate sunlight exposure prior to sediment deposition, limited post-depositional sediment mixing, and stable dose rate conditions. Partial bleaching is the incomplete solar resetting of a luminescence signal and it is typical of high-turbidity water columns [8], glacially-sourced sediment [9], or subaqueous reworking processes [4]. When overlooked, partial bleaching can lead to depositional age overestimation. Bioturbation can cause mixing of different-aged sediments and/or deviations from a stable dose rate environment over time, limiting the accuracy and precision of luminescence ages [1]. Pedogenic alterations, such as oxidation of iron and buried organic material, translocation of particles, dissolution and precipitation of evaporites, and shrink-swell processes, also lead to sediment mixing and dose rate heterogeneity. Sediment with clear signs of bioturbation or pedogenic alteration should be avoided for luminescence sampling. Beware that in sediment cores and under subdued lighting, these features may be difficult to identify. Disturbance from coring methods and subsurface extraction can cause additional mixing, uncertainty in dose rate conditions, and loss of the of the original luminescence signal. 

Subsurface samples have further complexity in their dose rate environment due to changes in groundwater levels and diagenetic processes. In the vadose zone, water content fluctuations impact the effective dose rate to which the sample was exposed, as water does not emit radiation and attenuates the dose absorbed by the grain [1]. Water saturation can also lead to mobilization of soluble radioelements and disequilibrium detected in the U-series decay chain, causing the dose rate environment to change over time [10]. Further complications in varying dose rate are found in lacustrine and marine settings where sediment compaction in the upper 5 m exponentially decreases sediment density and water content [11]. All of the factors mentioned here can impact the luminescence age estimate, and sampling details are provided for mitigating and controlling for unwanted sample material from sediment cores. 

## 2. Drilling and Augering Methods

Sediment character and site conditions will dictate the optimum auger or drilling setup and recovery. Sedimentologic considerations are burial depth, grain size (cohesion), and compaction (water content and induration) of the sediment being extruded, as well as site accessibility and driller/operator availability [12]. If solid core liners are available, then a light-proof (opaque) core liner/tube/barrel should be selected such as aluminum or steel (for deeper core depths, >7 m) or dark PCV or PE for shallower cores (1–2 m). For best sediment core recovery in subaqueous settings, the core length to core diameter ratio should be ≥6:1 to maintain enough internal friction to keep the core intact [13]. Larger diameter cores are preferred for luminescence sampling after extraction because of the greater volume of sediment available for dating after removal of the outer sediment adjoining the core liner. 

Generally, drilling method choice will depend on geologic setting, desired core length, and sample depth. Coring mechanisms well-suited for collecting samples specifically for luminescence dating include hand-augering (human or mechanized-power), vibracoring, sonic and percussion drilling, in addition to rotary drilling in limited use. Removal of the core or cuttings will be aided by a drill stem liner, core barrel, bucket, bailer, fluid, or air. For deeper cores that require flight extension and further penetration, casing, mud, or water may be used as means to stabilize the open borehole. Some of these methods may be combined or modified to fit specific sedimentologic, hydrogeologic, and depth objectives [13,14,15,16].

Key goals for successful coring and luminescence dating of core sediment are related to the preservation of original stratigraphy and the burial dose (natural luminescence signal). Sedimentary structures should be visible to help select the most suitable sediments for the D_E_ sample. Note that at least two D_R_ samples will be collected for each D_E_ sample (above and below) and undisturbed sediments are important for selecting intervals with intact sediments for all three sample intervals (two D_R_ and one D_E_ sample). In addition to D_R_ considerations, sediment disturbances can also mix different aged deposits. This can be minimized by limiting auger rotations or percussive drives. Minimizing loss of material during retrieval and post-extraction will also maintain the correct sample/core depth and allow accurate cosmic-dose contribution to the total D_R_ calculation. Recovery may be improved with the use of a vacuumed sample chamber (piston), or high internal friction (fine-grained sediment). Luminescence dating requires the sediment be kept in an opaque core liner, core-box, or bag until it can be subsampled in a darkroom laboratory. It is critical that the D_E_ sample be collected under safe lighting conditions, otherwise the sample is at risk of age underestimation due to loss of the natural signal [1]. We will discuss further methods for dealing with unlined and split (sunlight-exposed) cores in Section 3. 

### 2.1. Augers

Shallow-depth subaerial deposits (<10 m) can be sampled by using hand-augering (rotating bit and core barrel) in non-indurated sedimentary environments such as sand dunes, fluvial terraces, sandy soil, and loess. Compared to more powerful drilling rigs, soil augers are generally cost-effective, transportable to remote field sites, and some require only one person to operate [3]. Additionally, hand-auger coring systems are desirable for use in sensitive sites, where minimally invasive sample extraction methods are required. Soil augers typically use human power to advance the bit by rotating a T-shaped handle to which rod extensions are attached. These serve to extend the auger bit to the desired sample depth. The main disadvantages of soil augers are the length of core section (~0.3 m), limited depth range, and the destruction of sedimentary structures. The van der Staay suction corer is specifically designed to sample saturated sand up to 30 m depth, and intact core section lengths recovered range from 2.5 to 5 m [17]. More powerful motorized augers can achieve extraction at greater depths (60 m), particularly when a mechanized hydraulic pump is attached to the auger head and a powered hoisting apparatus assists drill stem removal [3]. Truck or track-mounted hollow stem augers are commonly used in geotechnical or water well drilling through unconsolidated sediments. The diameter of the stem ranges from about 6 cm to 15 cm, and each drive is ~1.5 m long. An inner liner painted black or split spoon corer may be placed inside the hollow stem to extract undisturbed core sections for luminescence dating.

#### Auger Sampling Methods for Sand Dunes

**Figure 1:**
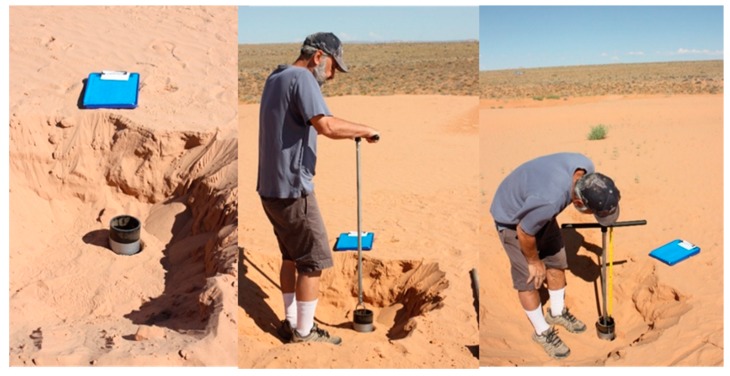
Hand-augering in Kanab Sand Dunes, southern Utah. Polyvinyl chloride (PVC) casing is inserted in top of auger hole to provide a reference platform for depth control and prevents influx of surface sediment downhole. The drill stem is advanced through the casing to the desired depth.

Shallow sand-rich deposits are often the preferred environment for luminescence dating applications, and methods for hand-augering in shallow sandy deposits are described in detail. The sampling site should be determined in the field where bioturbation (i.e., burrowing, root zones, human disturbance) can be avoided. Loose, non-cohesive surficial sediment should be removed to form a platform that reduces the potential for surface grains to fall into the auger hole. The thickness of any removed sediment should be noted to maintain accurate sample depth records. A PVC pipe can be placed at the auger location to act as a casing and prevent contamination of samples by surface sediment falling into the hole, as seen in Figure 1. This will also provide a reference platform to determine sample depth for both the D_E_ and D_R_ components in the OSL/IRSL sample.

**Figure 2:**
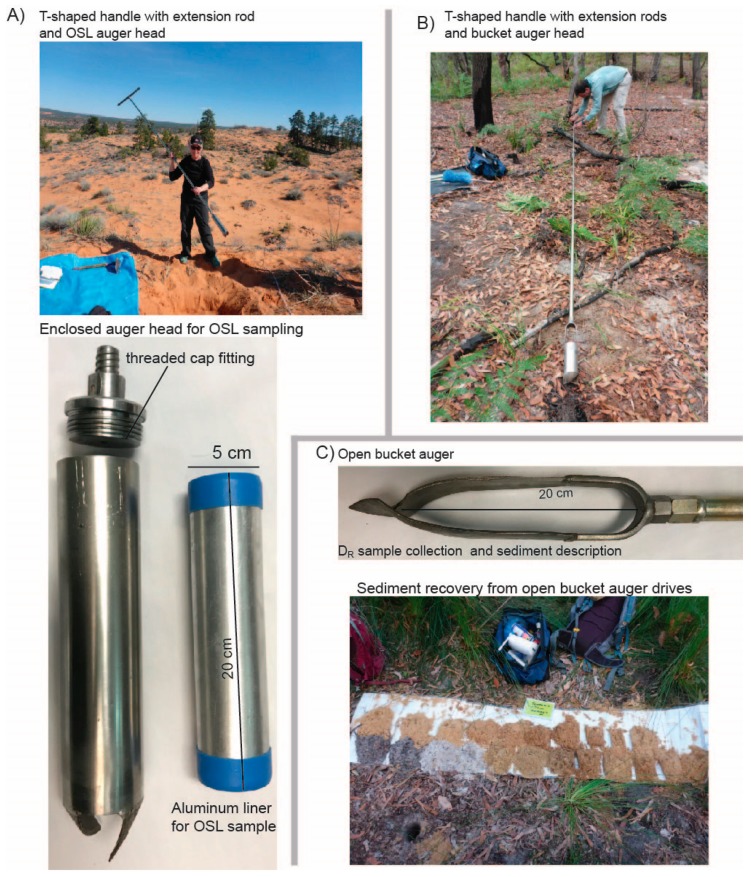
Shallow-sediment auger set-up for luminescence sampling. (**A**) Optically stimulated luminescence (OSL) auger head with T-shaped handle. The enclosed OSL auger head is fitted with an aluminum liner to contain the OSL sample in a light-proof tube. (**B**) T-shaped handle with extension rods are used to drive bucket auger. (**C**) Each drive requires sediment removal from hole, placed on the surface in stratigraphic order (by depth) until desired OSL sampling depth is reached. This material may be needed as part of the D_R_ sample.

Bucket augers can be used to collect sediment samples as the auger hole is advanced to greater depths during exploration. Various designs ranging from a closed bucket, as seen in Figure 2A,B, to an open catcher, as seen in Figure 2C, are available and the best choice will be dependent on the sediment characteristics and environmental setting. Each bucket auger drive should be saved in stratigraphic order (by depth) as it may be used for part of the D_R_ sample, as seen in Figure 2C. For dose rate sediment, collect ~300 g of material from the auger-drive(s) within 15 cm above and 15 cm below the OSL sample interval. When the target depth for the OSL sample is reached, switch out the bucket auger head for the OSL sample head, as seen in Figure 2A. Sample collection heads should contain an inner opaque liner into which the OSL sample can be captured without light exposure. Use of foam inserts keep the sediment packed while it enters the OSL tube and act as a barrier when the sediment reaches the end (top) of the OSL tube. Once extracted, the sampler is removed from the drill stem and the inner metal tube is capped on both ends to keep the sample intact and to prevent light exposure.

### 2.2. Vibracorers

Vibracoring utilizes steady and high-frequency vibrations to allow the sampler to move downward through subsurface deposits [13,18]. Vibracoring works best in fine-grained saturated sediments. Portability of vibracore devices can range from hand-held battery-operated corers to large vessel-mounted deep-water corers. Use of a steel core barrel is ideal for keeping the core light-proof for subsequent luminescence sampling. Undisturbed sediment cores greater than 10 m long may be obtained in finer-grained settings, while full recovery may be difficult in well-sorted water-saturated sands. Due to the vibration and rearranging of sediment particles, the amount of compaction should be calculated so that sample depth is accurate. For luminescence sampling of vibracored sediment, subsections of the core lengths may be cut and sent directly to the luminescence laboratory. Creation of a dark space is required if luminescence sampling occurs prior to shipment to the laboratory. Details for this in the field or remote dark room and subsampling are discussed below.

### 2.3. Piston and Gravity Corers

Gravity submersible and piston corers refer to similar coring methods and are gravity-driven, single-drive bottom penetrators. Obtainable core depth is based on the free-fall velocity and empirically calculated from water depth. Core length for gravity drivers is on the order of 1–5 m [12]. Piston corers can operate up to several hundred meters water depth depending on the drive mechanism [19]. The main advantage to piston-style corers is the partial vacuum created by the piston, which helps keep the sample intact [20]. Sediment does not enter the core barrel until the desired depth is reached [13]. These can operate via a rod or cables, noting that extension rods add weight and may limit the operational water depth [12]. Lake-bottom sediment corers are commonly used in limnological studies where preservation of stratigraphy and pollen are integral to sample analysis. The Livingstone-type drive rod piston corer is used for underwater operations and sediment sampling depths up to several meters are obtainable [21]. These techniques may be suitable for luminescence core sampling as well, preferably with core barrels and liners that are metal or black. If necessary, clear liners may be spray-painted black and core should be stored in a dark setting.

### 2.4. Percussion Drivers

Percussion drivers are a mechanized hammer that is restricted to vertical motion, typically used to advance the tube with core catcher into the sediment or rock by percussive force or direct push. These types of rigs are available as compact units for limited access situations up to industrial-sized track or truck-mounted rigs. For use in unconsolidated or semiconsolidated sediment above the water table, large diameter (15–25 cm) hollow-stem percussion drill rigs can drive through the subsurface to 30 m or more.

### 2.5. Rotary-Vibratory (Sonic) Drill

Sonic drilling combines rotary drilling and vibracoring mechanics in a dry drilling technique capable of extracting large diameter (30 cm) continuous core sections, up to 100 m depth. Core section length and recovery is dependent on the cohesiveness of drilled material. If clay content is high or the sediment is semi-indurated, then whole core sections on the order of 3 m per drive can be obtained [22]. For sonic drilling, the drill bit and auger stem are rotated as the auger is pushed downward [23], each flight is brought up to extrude the cored sediment. For luminescence dating, it is best for the extruded material to be stored in black plastic liners; however, the innermost core sediment may be sampled for the D_E_ even if cores and are stored in clear plastic liners if they have not been disturbed by liquefaction or desiccation, as discussed below.

### 2.6. Rotary Drill

Rotary drilling is essential for lithified or highly consolidated sediment and bedrock, and commonly produces rock cuttings which are not recommended for luminescence dating in most cases. Commonly, rotary drilling is used with water or mud to prevent borehole caving and to lift the cuttings out of the hole while the rotating auger head cuts through the rock. Borehole drilling that uses bentonite-based drilling additives should be avoided for luminescence dating. The major concern with type of additive is that it will be mixed with the dose rate material and will contribute an unknown amount of radiation to those measurements. Additionally, light-exposed grains or small clasts from further up the borehole could get mixed in the D_E_ sediment during retrieval and extraction.

Hydraulic rotary uses fluid circulation down the drill stem and return along the outside, while reverse rotary has fluid injected down the well and cuttings are brought up through the drill stem [24,25]. Each drive may be up to 10 m in length, allowing for drilling through several km of rock through either rod (conventional) or wireline extension methods [13]. Though not an endorsed application here, previous work has shown that cuttings may be large enough for luminescence characterization, particularly if there is a high content of silt and clay and drilling speed is reduced [26]. Telescoping casing can be used as a replacement for drilling mud to keep the borehole open, but at greater cost and decreasing core diameter with increasing depth [27]. An inner core barrel and special bit may be used if whole rock cores are desired for OSL dating or thermochronology [28]. OSL thermochronology is an innovative utility in neotectonics for quantifying rock exhumation and cooling rates through the Earth’s crust [29]. The concept for sampling remains the same, that is, the innermost material which has not been exposed to light is used for luminescence measurements.

## 3. Core Sampling Methods for Luminescence Dating

The methods outlined here discuss strategies for sample collection from cores and auger samples of differing integrity. First we discuss how to sample for OSL/IRSL dating on whole (unsplit) intact sediment core, weighing such factors as sedimentary and stratigraphic interpretation prior to luminescence sampling and keeping the core intact and light-safe prior to and during sampling. Considerations and protocols for OSL sampling of cores previously exposed to light will also be discussed. Ideally, two cores immediately adjacent to each other should be collected. The first will be the pilot core, which will be analyzed under normal lighting conditions to fully describe the sedimentary units and select target depths for the luminescence samples. The second core would then be extracted in a darkroom setting (or at least at the D_E_ sample target depths) such that intact samples can be extracted and sent to the luminescence laboratory of choice. The sediment core used for luminescence sampling should be kept in a light-proof casing and split (opened) under safe lighting conditions in a dark lab, as recommended below.

### 3.1. Whole (Unsplit) Cores

**Figure 3:**
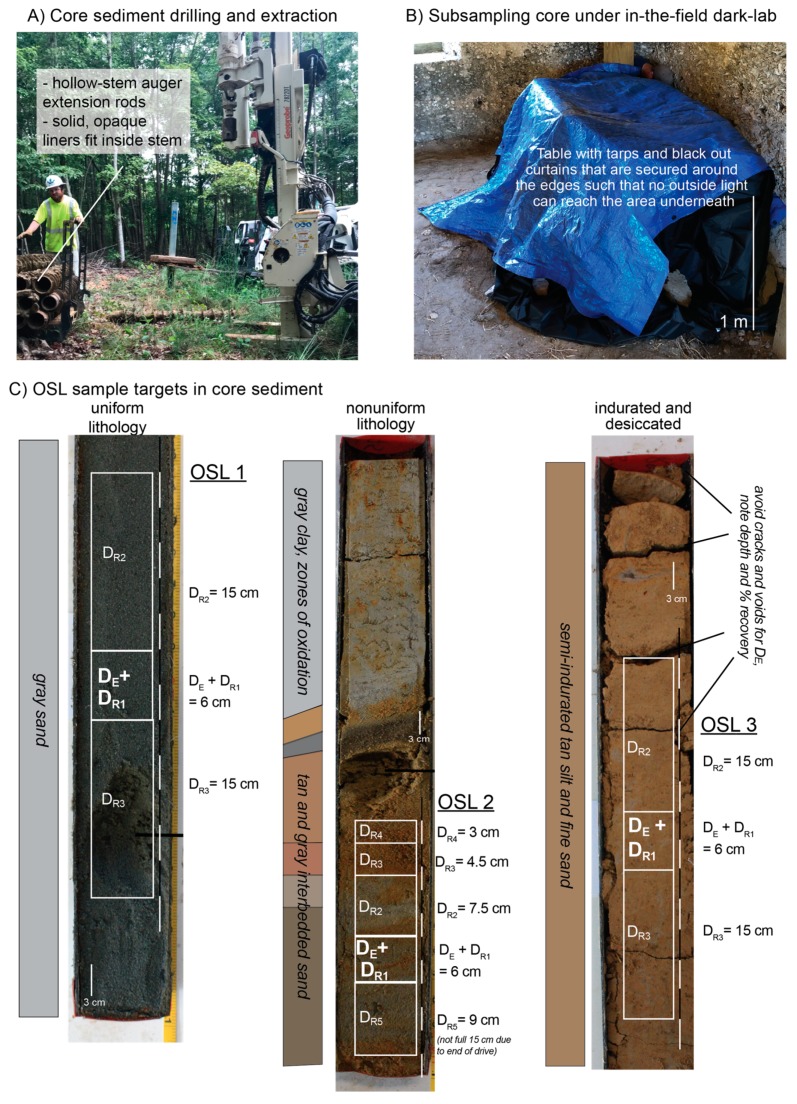
Whole core extraction and sampling with opaque liners. (**A**) Hollow-stem auger drill rig with flight extensions. Metal or black PVC tubing fits in opening for light-safe core sediment extraction. Photo credit: Abby Conklin-Muchnick. (**B**) Example of in-the-field dark space for subsampling core sediment. Tarps and blackout drapery are layered over folding table and edges are secured such that no outside light is visible under the material. Red LED headlamps can be used for lighting under the tarps. (**C**) Core photos of uniform, nonuniform sedimentology and stratigraphy, as well as indurated/desiccated core. Photo credit: Benjamin DeJong [16].

**Figure 4:**
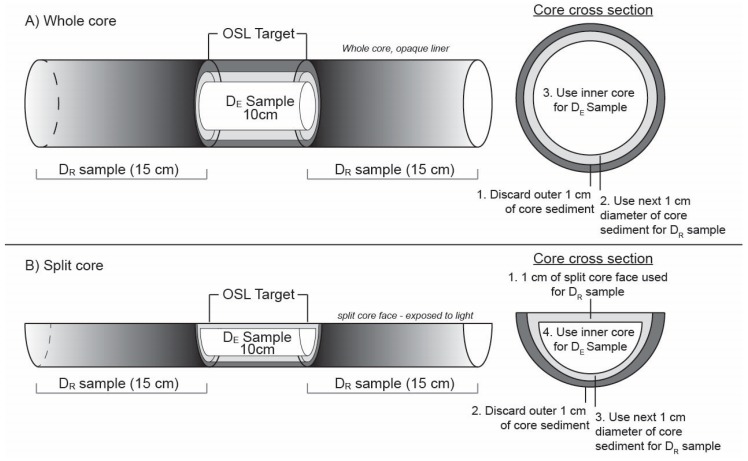
Idealized target sediment for D_E_ and D_R_ samples in luminescence dating applications to core sediment. (**A**) Whole core example preferably collected in opaque liner. The outer 1 cm is usually discarded as this may contain grains from unwanted sedimentary units above the target depth. The next 1 cm of core diameter used as dose rate sample for target depth. The innermost (light-proof) sediment is used for D_E_ sample processing. The overlying and underlying 15 cm of core is sampled for D_R_. Subsamples may be required if sedimentology is vastly different from the target D_E_ sample. (**B**) Split core example. If the core face has been exposed to light, 1 cm of sediment at the split core face is used for the D_R_ sample. The outer 1 cm rim of core material is typically discarded as in example A. The inner core is used for D_E_ sample processing. The overlying and underlying 15 cm of core is used as the D_R_ samples. Subsamples may be required if sedimentology is vastly different from the target D_E_ sample. Desiccation cracks in the core and zones of liquefaction should be avoided.

Whole or unsplit cores stored in light-proof core liners are the most suitable for luminescence dating of sediment and rock cores. Figure 3A shows an example of a hollow-stem auger system that can accommodate core liners. Transparent liners may be helpful in cases where a companion core is not possible, and the target sample range is only a few cm thick (i.e., single-event strata). Cores retrieved in transparent liners may be sampled for luminescence dating following proper sampling protocol to remove exposed grains on the outer perimeter of the core [30]. The PVC or PE liner and core may be cut into smaller section lengths at desired sample targets and sent to the luminescence laboratory for D_E_ sample extraction under subdued lighting conditions (see below). The length of the core section sampled for D_E_ and D_R_ will depend on several factors, as seen in Figure 3. First, the D_E_ sample should contain quartz and/or potassium feldspar-rich sand (>20% in the 63–250 µm range). The length of the core section sampled should ensure at least 10 g of datable sediment will make it through mineralogic processing. Roughly 2–10 cm intervals will suffice in larger diameter cores, as shown in Figure 3 and Figure 4, though greater length may be required in quartz and potassium feldspar-poor environments, such as evaporite rich or other fine-grained deposits. Water can be used to aid sample extraction in dry sands with little cohesion or indurated fine-grained sediment to ensure layers are removed uniformly and to help retain sample integrity. Unconformities should be avoided to help reduce the complexity of the dose rate environment and inclusion of different-aged depositional units in the D_E_ sample. The outer 1 cm of sediment in contact with the core liner should be removed prior to D_E_ sampling due to light exposure (in clear core liners) as well as the potential inclusion of sediment from different depths due to drag along the core liner. This outer material may be used for the D_R_ sample if there is minimal disturbance on the outer rim of the core. However, it should be discarded if there is evidence of friction and sediment smearing along the exterior of the core. Sampling for dose rate can become complex if multiple layers of varying grain size and/or lithology exist above or below the D_E_ sample. If different units are present within 15 cm above or below the D_E_ sample, each unit should be subsampled with distance to D_E_ sample noted, as seen in Figure 3C. A distance weighting will be used to average the radio-isotope contribution to the D_R_ because the sediments closest to the D_E_ sample will have a larger dose-rate contribution than sediments further away [1].

### 3.2. Opened Cores and Cuttings Exposed to Light

**Figure 5:**
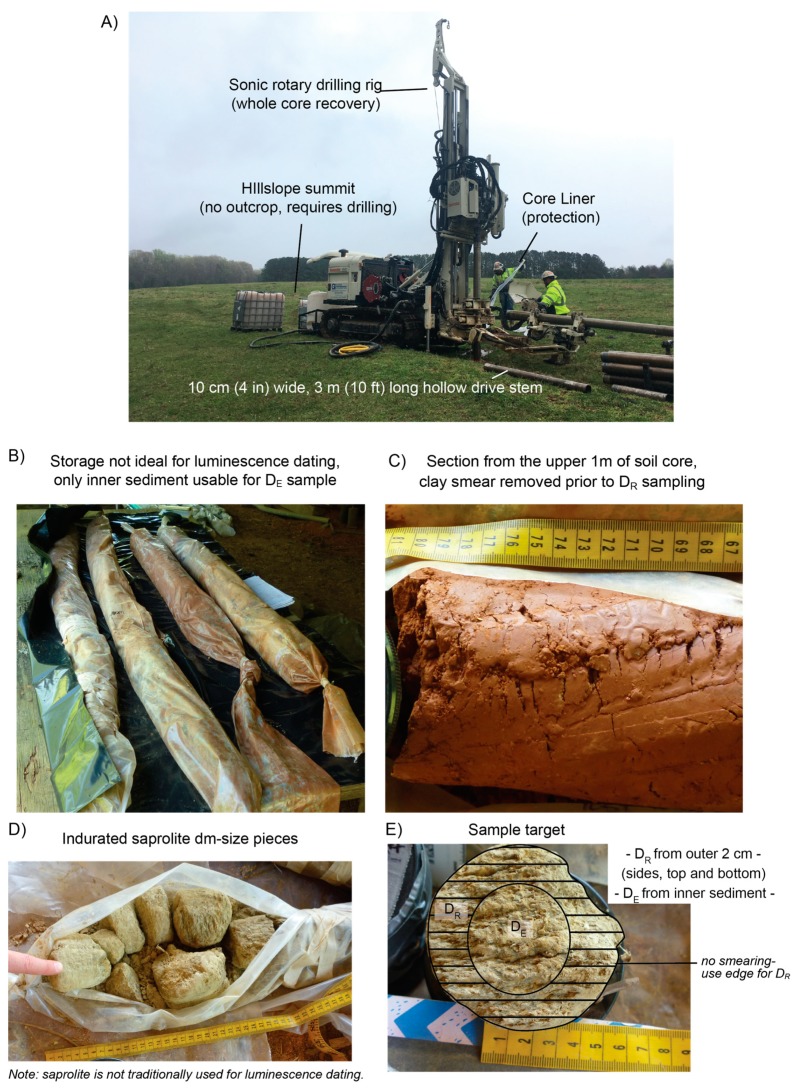
(**A**) Sonic-rotary drill rig at hillslope summit. Photo credit: Martha Eppes. (**B**) Yield, including intact soil and saprolite cores that were stored in plastic liners and outdoors. While not ideal, these conditions are not an impediment to luminescence geochronology [22]. (**C**) The upper 1 m of core shows signs of sediment and soil smearing on the sides, this material should be removed prior to dose rate sample collection. (**D**) Indurated saprolite, no smearing. (**E**) The dose rate sample will include the outer 1–2 cm from the sides, top, and bottom of the sample section. The inner most material is used for the equivalent dose sample.

Archived or stored sediment cores not originally intended for luminescence dating may also be sampled for OSL/IRSL if the following conditions are held. The core must remain in original stratigraphic order and depth should be known, along with percent of core recovery for proper cosmogenic dose rate calculation. If major desiccation has occurred while in storage, large cracks may form and can allow light to penetrate through the inner core and diminish the luminescence signal, as seen in Figure 3C. Additionally, the water content of a core in storage will not be representative of burial moisture conditions, a simple method for retrospectively estimating water content on desiccated core samples may be followed [31]. The outer 1–2 cm from each edge (exposed to light) will typically be removed in the dark lab, though it has been shown that <1 mm of sediment may be enough to shield the underlying material from light penetration in fine-grained sediments [4]. The exposed sediment can be added to the D_R_ sample material, which as noted above should come from units within 15 cm of the D_E_ sample, as seen in Figure 4B. When the outer core rim is contaminated with sediment from overlying units as indicated by smearing on outside of core, this material should be discarded. Recent exploratory work on soil and saprolite cores in the eastern US were successfully sampled for OSL/IRSL analyses [22], as seen in Figure 5A–E. For this work, three-meter sections of 10 cm diameter cores were extruded into clear plastic liners and stored outdoors. The methods for D_E_ and D_R_ subsampling included working with the core sections in the luminescence laboratory to:

1. Remove and discard 1–2 cm of clay smear from the outer diameter of the core.
2. Cut core in half to preserve one side for non-OSL analyses.
3. Collect sediment from the split face (inner material) of core for D_E_ processing, as seen in Figure 4B. In this case, the split core face material was safe to collect for the D_E_ sample as it was opened (split) in the dark room.
4. Collect remaining sediment for water content and D_R_ samples.

At times subsurface sediment and rock are collected in the form of cuttings and chips through rotary drilling. Although not ideal, if these materials are large enough, they can be utilized for luminescence dating. For example, 0.25–1 cm cuttings made available through boreholes drilled for a basin analysis study were used for luminescence characterization using IRSL [26]. Given the very limited amount of material available, full processing and mineralogic refinement to isolate potassium feldspar or quartz was not feasible. Additionally, after removing the outer few mm of exposed grains there was not enough material to make the chemical measurements needed for dose rate calculation and thus apparent ages were reported [26].

### 3.3. In-the-Dark Field Sampling and Portable Luminescence Measurements on Core

Night-time subsampling or the construction of a field-based or off-site dark-room shelter may be required in some situations, for example if the core is needed for other analyses, or to reduce shipping costs. If night-time working conditions are not possible or suitable due to site restrictions based on safety, access or artificial lighting then cores should be taken to a windowless room in a building that is large enough to accommodate the core sections. For more remote field sites, a tent or some similar structure such as a very large cardboard box or folding table will suffice. This will support the layers of tarps and blackout curtains and sheeting needed to create the condition of total darkness required for selecting the luminescence samples, as seen in Figure 3B. Note that it can be ~5 °C (10–15 °F) warmer with little airflow under the blackout materials, so caution should be taken under extreme heat. 

Once under night-time or darkroom conditions, only light sources with wavelengths of amber (~590 nanometers, nm) and red (~700 nm) light should be used to illuminate the work area. All other wavelengths of light, particularly those with shorter wavelengths (blue and white light or sunlight) will rapidly destroy the luminescence signal stored in the sediment. Additionally, near-infrared and infrared light sources will remove the luminescence signal stored in potassium feldspar (~890 nm). Common safe-light sources include red bike lights (only those with red light-emitting diodes (LEDs)). White LEDs with a red filter are not recommended for this purpose. It is important that these lights are only used as indirect light-sources and not directly shined on to the sediment core. Alternatively, if power is available at the sample site, a shop light with a red lightbulb (8 watt) can be used. Again, this light source should not be directed on the sediment core and should serve as an ambient light source for the space. It not necessary for the D_R_ sample to be collected under dark conditions, unless there is concern some D_R_ material might be needed for D_E_ processing due to a limited amount of datable mineral grains in the sample.

A well-designed, yet underutilized tool for rapid continuous-core stratigraphic analysis is the scanning luminescence reader [32]. The reader and sediment core are placed under total darkness, and the core moves under the photomultiplier reader by a motorized bed. The natural luminescence signal is measured along the entire length of the core [32], with an output similar to a gamma log. Advantages to this unique whole core analysis include: (1) it is nondestructive such that other analysis can take place after it is performed; (2) it can aid visualization of stratigraphic breaks at the mm scale; and (3) it displays zones with higher luminescence intensity to indicate better sedimentary targets for luminescence sampling (or those deposited under poor bleaching conditions as they too can have large luminescence response to stimulation) [32]. Note that this apparatus must be constructed individually. To our knowledge, it cannot be purchased as an assembled piece of equipment and has no professional installation option, requiring some knowledge of basic electrical engineering, blue or IR LEDs, a photomultiplier tube, a motorized core bed table (5 mm/sec), and sensor cards.

A portable luminescence reader has recently come on the market [33]. The portable unit utilizes much smaller sample sizes than those used in traditional luminescence dating, provides rapid luminescence measurements, and may be transported to core storage facilities. High-resolution subsampling (i.e., every 5–10 cm) of sediment cores can be run through the portable luminescence reader to rapidly identify stratigraphic changes and sedimentary targets to sample for full luminescence analysis. These measurements must be carried out in a darkroom facility if used on site.

## 4. Uncertainties and Mitigations

The systematic uncertainties in luminescence dating of core sediment are the same for noncore OSL/IRSL samples and are related primarily to instrument calibration and dose rate conversion factors [1]. Random uncertainties are sample specific and include pre-, syn- and post-depositional processes, OSL/IRSL and radioelement measurement uncertainties and sampling errors. For this reason, the random error on OSL/IRSL samples collected from cores may be greater than for traditional samples collected from well-exposed and carefully selected outcrop settings.

One contribution to D_E_ uncertainty is partial bleaching of a previously acquired burial dose that was not fully reset prior to deposition [34]. Poor luminescence zeroing can cause age overestimation and occurs in high-energy or extremely turbid depositional environments, such as subaqueous slumping, marine storm deposits, alluvial flood packages, proglacial, and subglacial deposits where sediment concentration is high and light penetration is low. Single-grain dating [35] and use of advanced statistical methods such as the Minimum Age Model [36] mitigate this effect by helping to identify the population of individual-grain D_E_ values that were fully reset by sunlight. Additional contribution to D_E_ uncertainty occurs with post-depositional mixing of different-aged sediment through liquefaction and resuspension of benthic sediments, bioturbation [37], and soil processes [38]. These features are often identifiable through detailed sedimentology (i.e., krotovina identification), chronostratigraphy (i.e., age reversals) and D_E_ statistics (i.e., overdispersion, skewness). Other sources of D_E_ uncertainty are inherent to the sediment and are less impacted by core sampling methods, including micro-dosimetry, and differences in luminescence sensitivity and signal components between grains from the same sample.

Dose rate uncertainty can be more complex for OSL/IRSL samples collected from sediment core than those collected from outcrop exposures. Water content in sediment pore spaces plays a key role in radiation attenuation, that is, as water content increases the dose rate is effectively reduced [1]. Samples in the vadose zone often have highly variable annual water content due to seasonal fluctuations in the water table, as well as longer-term climate-related factors. Additionally, soil-moisture retention factors like aspect, vegetation type, canopy cover, and geology play a key role in water content. *In situ* moisture content can be representative of average burial water content conditions; however, it is also likely that *in situ* samples over or underestimate average moisture conditions if there has been a recent precipitation event or long period of no precipitation. Additionally, sediment compaction will reduce pore spare and lower the *in situ* water content over time. Mean water state can be projected from soil moisture classification mapping to select value(s) of average water content for use in the D_R_ calculation [39]. Deposition and erosion impact paleo-moisture conditions by driving changes in base-level. Moisture samples from exhumed deposits, or coastal environments that have been through multiple cycles of marine transgression and regressions may not be representative of mean-state moisture content and might require a retrospective approach to estimate water content [16]. Dose rate modelling is required in cases like these or where a single value will not effectively represent the complex moisture history of a subsurface sample.

Further uncertainties in the dose rate measurements and calculation from sediment cores can arise from the presence of organic material, solubility of minerals, and pedogenesis. In the presence of large quantities of buried organic material, radioactivity is absorbed and attenuated [40]. For saturated sediments, decay chain disequilibrium can be an issue when radioelements are water soluble and mobilize, creating chemical excesses or deficits that do not reflect past burial chemistry [41]. Soluble minerals (i.e., calcite, halite) can precipitate out of solution in the vadose zone and change the overall dose rate environment over time, usually lowering the dose rate as they are not highly radioactive [42,43]. Additionally, pedogenic processes such as eluviation (leaching) and translocation add uncertainty in dose rate calculations for soil samples as deep weathering will chemically alter and physically move soluble and reactive elements and minerals down profile [44].

## 5. Summary

Sediment cores are essential for reconstructing Quaternary geologic history, and luminescence dating is at the forefront as a commonly used tool for building such geochronologies. Luminescence dated core sequences are used in (but not limited to) reconstructing flood chronologies, shoreline development, sea level change, dune field evolution, soil development, basin analysis, and paleoseismology. Coring mechanisms and devices are more readily available, and some are novice-user-friendly, in addition to being more compact for use in remote and ecologically-sensitive sites. It is possible to date sand or silt in previously collected sedimentary cores in storage using OSL/IRSL, given a few conditions are met. Knowledge of the basic uncertainties outlined here should help investigators target better D_E_ and D_R_ samples to meet the growing demand for better age resolution and more precise geochronology from sediment cores.
